# Characterization of *Salmonella enterica* from invasive bloodstream infections and water sources in rural Ghana

**DOI:** 10.1186/s12879-018-2957-4

**Published:** 2018-01-19

**Authors:** Denise Dekker, Ralf Krumkamp, Daniel Eibach, Nimako Sarpong, Kennedy Gyau Boahen, Michael Frimpong, Elina Fechtner, Sven Poppert, Ralf Matthias Hagen, Norbert Georg Schwarz, Yaw Adu-Sarkodie, Ellis Owusu-Dabo, Justin Im, Florian Marks, Hagen Frickmann, Jürgen May

**Affiliations:** 1 Infectious Disease Epidemiology, Bernhard Nocht Institute for Tropical Medicine (BNITM), Bernhard-Nocht-Str. 74, D-20359 Hamburg, Germany; 2 German Center for Infection Research (DZIF), Hamburg-Borstel, Lübeck, Germany; 3 Kumasi Centre for Collaborative Research in Tropical Medicine (KCCR), Kumasi, Ghana; 4 Bundeswehr Hospital of Hamburg, Germany, Department of Tropical Medicine at the Bernhard Nocht Institute, Bernhard-Nocht-Str. 74, D-20359 Hamburg, Germany; 5 Kwame Nkrumah University of Science and Technology (KNUST), Kumasi, Ghana; 6 Epidemiology Unit, International Vaccine Institute (IVI), Seoul, Republic of Korea; 7 The Department of Medicine, The University of Cambridge, Cambridge, UK; 8 Institute for Medical Microbiology, Virology and Hygiene, University Medicine Rostock, Schillingallee 70, 18055 Rostock, Germany

**Keywords:** invasive non-typhoidal *Salmonella*, water sources, transmission reservoir

## Abstract

**Background:**

Non-typhoidal *Salmonella* (NTS) cause the majority of bloodstream infections in Ghana, however the mode of transmission and source of invasive NTS in Africa are poorly understood. This study compares NTS from water sources and invasive bloodstream infections in rural Ghana.

**Methods:**

Blood from hospitalised, febrile children and samples from drinking water sources were analysed for *Salmonella* spp. Strains were serotyped to trace possible epidemiological links between human and water-derived isolates.. Antibiotic susceptibility testing was performed,

**Results:**

In 2720 blood culture samples, 165 (6%) NTS were isolated. *S.* Typhimurium (70%) was the most common serovar followed by *S.* Enteritidis (8%) and *S.* Dublin (8%). Multidrug resistance (MDR) was found in 95 (58%) NTS isolates, including five *S.* Enteritidis. One *S.* Typhimurium showed reduced fluroquinolone susceptibility. In 511 water samples, 19 (4%) tested positive for *S. enterica* with two isolates being resistant to ampicillin and one isolate being resistant to cotrimoxazole. Serovars from water samples were not encountered in any of the clinical specimens.

**Conclusion:**

Water analyses demonstrated that common drinking water sources were contaminated with *S. enterica* posing a potential risk for transmission. However, a link between *S. enterica* from water sources and patients could not be established, questioning the ability of water-derived serovars to cause invasive bloodstream infections.

## Background

*S. enterica* causes more than 1.2 million annual deaths worldwide, the majority occurring in resource-poor countries [1]. *Salmonella* infections other than typhoid fever, so-called non-typhoidal *Salmonella* (NTS), are usually limited to gastrointestinal disease in industrialized countries. In contrast, in sub-Saharan Africa (SSA), NTS are one of the most frequent causes of bacterial bloodstream infections in both adults and children, associated with high case fatality rates of 20 to 47%, also in Ghana [2–5]. In industrialized countries, infections with NTS are typically of zoonotic origin with regular food-borne outbreaks being described [6–8]. A broad spectrum of animal products such as poultry, beef, pork and eggs as well as contact to farm animals have been associated with infections [9–11]. The *Salmonella* serovar Enteritidis has been strongly linked to poultry farming and egg production [12].

So far, studies from SSA on *S. enterica* isolated from livestock and animal products demonstrate a broad *Salmonella* serovar distribution of types not commonly associated with human infections hence suggesting other transmission routes [13–15]. Despite the disease burden caused, the exact mode of transmission of invasive non-typhoidal *Salmonella* (iNTS) is largely unknown.

Residents in many SSA regions often do not have access to safe drinking water, but use water from sources such as rivers, lakes, wells and boreholes, which may be contaminated with bacteria from environmental sources, such as sewage wastewater in the absence of good sanitary facilities.

There is evidence that *Salmonella* serovars are specifically adapted to the human host with no or limited potential to be transmitted beyond this reservoir, suggesting anthroponotic transmission as a major route of recently evolved African strains [16–18]. So far, comparisons on serovar level of human and environmental *Salmonella* isolates from SSA have rarely been conducted. This information is important to understand reservoirs, and potential transmission routes of iNTS in order to institute efficient management and control strategies.

In the rural Asante Akyem district in Ghana, we investigated contamination of drinking water with *S. enterica* to identify a potential source for strains causing invasive blood stream infections in hospitalized febrile children.

## Methods

### Study site and laboratory procedures

The study was conducted in the rural Asante Akyem District in Ghana, which is the catchment area of the Agogo Presbyterian Hospital (APH), a district hospital with 250 beds. The municipal area has an estimated population of 142,400 inhabitants, spread over an area of 1160 km^2^. The region has a tropical climate with two rainy seasons from March to June and from September to October and is mainly covered by secondary rain forest and cultivated land. Malaria is highly endemic in this area.

Blood was taken from children aged ≤15 years attending APH with fever (≥38 °C) between September 2007 and November 2012. For microbiological analysis, 1–3 ml venous blood was injected into vials for paediatric blood cultures (Becton Dickinson, NJ 07417, USA) and incubated in an automated BACTEC 9050 instrument (Becton Dickinson). Broth from positive blood culture bottles was examined microscopically (Gram stain) and plated on MacConkey agar, Columbia agar enriched with 5% sheep blood, and chocolate agar (Oxoid, Hampshire, United Kingdom). The following organisms were classified as contaminants: coagulase-negative *Staphylococcus* spp.*, Micrococcus* spp., *Propionibacterium* spp., coryneform bacteria and *Bacillus* spp.

Water samples were collected from 69 villages within the Asante Akyem from October 2009 until December 2009. Water sources considered for sampling were those commonly used by the village populations to collect drinking water, namely wells, rivers, boreholes, outdoor pipes and container-stored water from unknown origin. From the collected water samples, 100 ml was filtered with a 0.45 μm pore cellulose membrane filter (Millipore, Cork, Ireland). The filter was placed into an enrichment broth (Selenite F broth, Oxoid), which was further sub-cultured onto a chromogenic medium (Brilliance *Salmonella* agar, Oxoid) after 18–24 h incubation at 35–37 °C in normal atmosphere. For the identification of *Salmonella* spp*.*, the Analytical Profile Index (API 20E) test (bioMerieux, Durham, North Carolina) was performed and confirmed by a *Salmonella* Latex Test (Oxoid). Serotyping was carried out with standard antisera (SIFIN, Berlin, Germany) according to the White Kauffmann le Minor Scheme. For *Salmonella* positive samples, two colonies were selected to increase the chance for detecting multiple serovars per sample.

### Antibiotic susceptibility testing

Susceptibility testing was performed using the disk diffusion method (Kirby Bauer) and interpreted using current Clinical and Laboratory Standards Institute (CLSI) guidelines (www.clsi.org). *Salmonella* isolates were tested for the following antibiotics: ampicillin, ampicillin/sulbactam, ceftriaxone, chloramphenicol, nalidixic acid, cotrimoxazole and tetracycline. Minimum inhibitory concentrations (MICs) for ciprofloxacin were determined by E-test (Oxoid). Isolates were interpreted as ciprofloxacin susceptible with an MIC ≤0.06 μg/mL, as intermediate (reduced susceptibility) with an MIC < 1 μg/mL and > 0.06 μg/mL and as resistant with an MIC ≥1 μg/mL. Ceftriaxone was used as a screening drug for the detection of extended spectrum beta lactamase (ESBL) producing strains. *Salmonella* isolates exhibiting resistance to ampicillin, cotrimoxazole, and chloramphenicol were classified as multidrug resistant (MDR).

### Statistical analysis

Descriptive statistics were applied to show variable distribution amongst blood and water samples. Observations with missing values were not excluded from the analysis, thus possibly resulting in different denominators. Results were presented for blood and water samples separately and were finally compared. All analyses were conducted using Stata Statistical Software 14 (College Station, TX: StataCorp LP).

## Results

### Bacterial bloodstream infections

Blood culture samples were collected from 2720 patients of whom 1255 (45%) were females. Median age of all study children was 2 years (IQR: 0–4) and children positive for *S. enterica* had a median age of 2 (IQR: 1–3). Two hundred forty-one (9%) positive blood cultures were classified as contaminants and excluded from the analysis. Pathogenic bacteria were isolated from the remaining 382 (14%) positive blood cultures, with *S. enterica* being the most frequently detected bacterial species (*n* = 222, 58%). Within *S. enterica,* 165 (43%) NTS and 57 (15%) *S.* Typhi were isolated. The three most common NTS serovars were *S.* Typhimurium (*n* = 115; 70%), *S.* Enteritidis (*n* = 13; 8%) and *S.* Dublin (*n* = 8; 5%; Table 1).

**Table 1:** Non-typhoidal *Salmonella* serovars and multidrug resistance in children attending Agogo Presbyterian Hospital, Ghana

NTS serovars	Frequency (%) *N* = 165	MDR n/N (%)
*S*. Typhimurium	115 (70)	76/113 (67)
*S*. Enteritidis	13 (8)	0/12 (0)
*S.* Dublin	8 (5)	1/6 (17)
*S*. Heidelberg	1 (1)	0/1 (0)
*S*. Rostock	1 (1)	0/1 (0)
*S*. Stanleyville	1 (1)	0/1 (0)
*S*. Virchow	1 (1)	0/1 (0)
Other serovars^a^	25 (15)	18/28 (64)

*Abbreviations*: *NTS* non-typhoidal *Salmonella*, *MDR n/N* Multi-drug resistance in ampicillin, cotrimoxazole and chloramphenicol (data not available for all samples)

^a^serovar unknown due to loss of isolate

### Antimicrobial susceptibility

Ninety-five (58%) NTS strains exhibited MDR (Table 2). All strains were sensitive to ceftriaxone, thus testing for ESBL-producing *Salmonella* strains was not performed. Reduced ciprofloxacin susceptibility was confined to five *S.* Enteritidis and one *S.* Typhimurium strain.

**Table 2:** Contamination of water samples with *Salmonella enterica* collected in the Asante Akyem district, Ghana

Water Source	Samples analysed	*Salmonella* contamination (%)^a^
Well	249	10 (4.0)
Household^b^	136	1 (0.7)
Borehole	60	0 (0.0)
River	55	8 (14.5)
Pipe	11	0 (0.0)

^a^percentage per samples analysed

^b^container-stored water of unknown origin in households

### Water analysis

The majority of water samples were collected from wells (*n* = 249; 49%), followed by container-stored water of unknown sources (*n* = 136; 27%) (Table 2).

*S. enterica* was isolated from 19 (4%) water samples. While samples from rivers had the highest *Salmonella* contamination (n = 8; 15%), no *Salmonella* were isolated from pipe or borehole samples. Amongst the 19 *Salmonella* positive water samples, 22 *Salmonella* isolates were identified. Three of the samples contained two different *Salmonella* serovars. In total, 14 different serovars were found including the following: *S.* Ajiobo (n = 1), *S*. Colindale (n = 2), *S. Corvallis* (n = 1), *S*. Duisburg (*n* = 3), *S*. Georgia (n = 1), *S*. Kingston (n = 1), *S*. Mim (n = 1), *S*. Poona (n = 1), *S*. Pramiso (n = 1), *S*. Rovaniemi (n = 1), *S*. Pasing (n = 1) *S*. Rubislaw (n = 3), *S*. Santander (*n* = 4), and *S*. Stanleyville (n = 1). Apart from two ampicillin and one cotrimoxazole resistant isolate, all isolates were susceptible to all tested antibiotics.

There was no overlap between the water-derived *Salmonella* serovars and the iNTS serovars.

## Discussion

The results highlight the significance of MDR *S. enterica* as a major cause of bacterial bloodstream infections in children in rural Ghana and emerging FQ resistance primarily related to *S.* Enteritidis. The study demonstrates a distinct distribution of *Salmonella* serovars with no overlaps between human and water-derived samples. Hence, *Salmonella* frequently found in drinking water are probably not a major source for invasive bloodstream infections in humans. Recent studies from SSA, using Whole Genome Sequencing methods, strongly suggest that *Salmonella* serovars causing invasive infections in humans have evolved and adapted within specific hosts [16–19]. These data support the hypothesis that invasive *Salmonella* infections are rather transmitted within the human population and not originate from zoonotic sources and are therefore less frequently found in the environment.

In addition, improved awareness of gastrointestinal infections and hygiene practices in the study area, might explain the infrequent environmental contamination with human *Salmonella* strains.

Currently, little information is available from resource poor countries on contamination of *Salmonella* serovars in water sources, although studies have shown the presence of a large diversity of different serovars in the aquatic environment [20–23]. The data correlates well with previous studies showing that rather unusual serovars, not-typically encountered in clinical specimens, colonise drinking water sources. This study indicates that contamination with *S. enterica* is frequent in the Asante Akyem District especially in dug well and river water. Animals such as reptiles may play an important role in the contamination of water sources, as these are known to be carriers of a vast variety and of uncommon serovars [24]. Overall, data on the potential of such strains to cause disease is scarce and was not investigated in this study. However, environmental *S. enterica* strains might play a significant role in self-limiting gastrointestinal infections not resulting in invasive disease with hospital admissions. Nonetheless, as no stool samples were assessed, this hypothesis remains speculative. Still, it is known that *S. enterica* found in drinking water may constitute a risk to human health because almost all serovars of *S. enterica* have the potential to cause illness in man [20].

Furthermore, resistance to locally administered antibiotics was high amongst *S. enterica* from blood cultures but almost absent amongst isolates from water. This suggests that *S. enterica* from water samples were not previously or repeatedly exposed to selective drug pressure as a result of previous antimicrobial treatment.

As for *Salmonella* blood culture isolates, reports across the African continent from studies with similar inclusion criteria have been published, in which the predominance of MDR *S. enterica*, in particular infections with NTS have been reported [25, 26]. The high frequency of MDR *S.* Typhimurium and *S.* Enteritidis in the study presented here confirms the distribution described in the review by Reddy and colleagues [3].

Drawbacks of the study include different collection times of water and blood culture samples complicating interpretation of transmission pathways. In addition, only two individual colonies were selected per sample while several distinct serovars may colonize one water source at the same time. This may have decreased the chance of detecting multiple serovars in one source and hence possible associations. Also, the overall test sensitivity would have increased by testing larger amounts of water and by longitudinal testing. Nevertheless, the *Salmonella* isolates found in the water samples give a crude estimation of the serovar composition of prevailing strains in the aquatic environment in the study area. Although the sampling strategy cannot be considered as representative, the exemplarily testing at least demonstrates that serovars found in invasive human disease do not play a quantitatively dominating role in local water sources. It, however, remains speculative where the *Salmonella* serovars found in the water sources predominantly come from and what their potential to cause disease is. These are important questions to be further investigated.

## Conclusion

Quantitative relevance of water-associated transmission of iNTS seems unlikely in this study area. Nevertheless, water contamination with *S. enterica* might play a role in gastrointestinal infections, which should be further examined.

There is an important information gap, which needs to be filled to understand infection reservoirs and transmission pathways of iNTS in order to devise effective management and control strategies. Future studies are required that focus on genome comparisons of human and zoonotic iNTS isolates in order to investigate *Salmonella* adaptation to the host more thoroughly and possible anthroponotic transmission.

Also MDR and emerging fluorquinolone resistance in *S. enterica* associated bloodstream infections in children from SSA urge to investigate evidence-based preventive interventions, like hygiene and sanitation measures or vaccines for high-risk populations.

## Abbreviations

APH: Agogo Presbyterian Hospital
API: Analytical Profile Index
BNITM: Bernhard Nocht Institute for Tropical Medicine
CLSI: Clinical Laboratory Standards Institute
DNA: Deoxyribonucleic Acid
ESBL: Extended spectrum beta lactamase
FQ: Fluorquinolone
iNTS: invasive nontyphoidal *Salmonella*
IVI: International Vaccine Institute
KCCR: Kumasi Centre for Collaborative Research in Tropical Medicine
KNUST: Kwame Nkrumah University of Science and Technology
MDR: multidrug resistance
MICs: minimum inhibitory concentrations (MICs)
NTS: non-typhoidal *Salmonella*
PFGE: Pulsed-field gel electrophoressis
SSA: sub-Saharan Africa
